# Neurodegeneration biomarkers in Alzheimer’s disease: axonal density index expands the “N” in the AT(N) framework

**DOI:** 10.1172/JCI202411

**Published:** 2026-02-02

**Authors:** Ryn Flaherty, Arjun V. Masurkar

**Affiliations:** 1 Aix Marseille Université, CNRS, CRMBM, Marseille, France.; 2 Center for Cognitive Neurology, Department of Neurology, and Department of Neuroscience, NYU Grossman School of Medicine, New York, New York, USA.

## Abstract

Neurodegeneration, along with amyloid and tau, define the AT(N) framework of Alzheimer’s disease that has shaped the development of diagnostics and therapeutics. Yet, biomarker development for neurodegeneration has lagged behind that for amyloid and tau, with limited definition of its heterogeneous microstructural aspects that may each serve as critical measures. In this issue of the *JCI*, Gong et al. leveraged diffusion MRI to derive a unique measure of axonal injury or axonal density index (ADI). Through cross-sectional and longitudinal analyses, they demonstrated that the ADI has superior performance in detecting, tracking, and predicting clinical impairment compared with prior diffusion MRI methods to evaluate axonal health and standard biomarkers of amyloid and tau. As such, the ADI measure may serve as an important expansion of the neurodegeneration biomarker repertoire.

## Introduction

The rising epidemiologic burden of Alzheimer’s disease (AD) and related dementias necessitates the development of more comprehensive biomarkers to characterize disease pathophysiology in vivo to better identify at-risk individuals and further develop disease-modifying therapies. The current working hypothesis for AD pathophysiology is based on the sequential development of amyloid, tau, and neurodegeneration, referred to as the AT(N) framework, which has shaped diagnostic approaches and therapeutic development (1, 2). The temporal evolution of AT(N) parallels the progression of clinical phenotype from preclinical (cognitively normal) to prodromal (mild cognitive impairment) to the dementia stage of AD. Currently, AT(N) can be monitored via imaging, cerebrospinal fluid (CSF) assays, and blood-based measures. Imaging is the preferred modality given its ability to quantify the extent and spatial distribution of pathology directly in the brain, with amyloid and tau burden measured via PET and neurodegeneration quantified via structural MRI.

## Limitations of current methods assessing neurodegeneration in AD

Within the AT(N) framework, neurodegeneration is a critical biological endpoint indicating the culmination of amyloid- and tau-mediated damage and the development of clinical symptoms. Typically, neurodegeneration is assessed using structural MRI, with macroscale measurements of medial temporal volume loss and cortical thinning (2). However, these macrostructural changes may be the result of many microstructural changes, including axon loss, neuronal or glial cell body loss, and dendritic pruning (3). Measures of volume loss and cortical thinning also fail to account for changes in white matter, which are increasingly implicated in early AD (4). Such microstructural white matter changes may have unique predictive capabilities and could not only reflect diverse pathophysiological ramifications of amyloid and tau, but also other processes increasingly recognized as contributing to AD, namely, inflammation and cerebrovascular dysfunction (2). Thus, it is critical to refine imaging methods that can evaluate these changes in early AD.

## Diffusion MRI–based microstructural measurements of axonal injury

Diffusion MRI has the potential to sensitively measure axonal injury in the white matter across disorders. Most work on imaging-based markers of axonal injury has been conducted in traumatic brain injury (5) and, for more advanced diffusion MRI models, in multiple sclerosis (6). In these disorders, diffusion MRI is generally more sensitive than traditional structural MRI, including both quantitative measures using diffusion tensor imaging and qualitative expert reading of apparent diffusion coefficient maps (5, 6). Diffusion tensor imaging measures of axonal injury also correlate well with CSF measurement of neurofilament light chain, a biofluid marker of axonal damage (7, 8). Research in multiple sclerosis has shown that microstructure models of diffusion MRI, such as neurite orientation dispersion and density imaging (NODDI) and standard model of imaging (SMI), can outperform diffusion tensor imaging measures of axonal injury (6). Microstructure models work by parsing the diffusion MRI signal into different compartments, often including intra-axonal water, extra-axonal water including extracellular matrix and glia, and free water including CSF (9). They can thus be used to generate an axon density index (ADI), which is the fraction of intra-axonal water compared with the total water volume. However, these measures of ADI become less reliable in the presence of high free water fraction (10), which is particularly an issue in neurodegenerative disorders such as AD (11). Despite this, there has been little research investigating the impact of free water correction techniques on ADI measures in AD. Further, there are limited longitudinal studies on ADI in AD.

## Constrained NODDI measurements of ADI better predict AD

In this issue of the JCI, Gong et al. (12) leveraged the Alzheimer’s Disease Neuroimaging Initiative (ADNI) dataset to conduct a study of whole white matter ADI derived from a free water–corrected implementation of NODDI called constrained NODDI (C-NODDI), which they propose to be a more physiologically relevant measure of axonal health. Instead of using the diffusion MRI signal to estimate the free water fraction as done in the original NODDI implementation, C-NODDI estimates the free water fraction by segmenting a structural T2-weighted image using the FMRIB Software Library Automated Segmentation Tool (8). They subsequently compared the performance of their C-NODDI ADI metric with metrics derived from previous measures of axonal density, namely, NODDI and SMI, in a cohort of participants that were cognitively normal and cognitively impaired (comprising mild cognitive impairment and AD dementia).

They first demonstrated that their measure of ADI differentiated between subjects that were cognitively impaired and cognitively normal at baseline, with those that were impaired showing significantly lower C-NODDI measures of axonal density. Moreover, subjects with cognitive impairment showed a significantly greater longitudinal decline in ADI compared with those that were cognitively normal. They subsequently found that baseline C-NODDI ADI successfully predicted longitudinal cognitive and functional outcomes in the cognitively impaired group. The longitudinal decline in C-NODDI ADI also tracked with declines in cognition and function in this group. Importantly, all of the above findings were valid even when the impaired cohort was restricted just to participants with mild cognitive impairment. Furthermore, C-NODDI outperformed NODDI and SMI across detection, prediction, and tracking of clinical impairment.

They next evaluated ADI against standard biomarkers of proteinopathy. In cognitively impaired participants, the capability of C-NODDI ADI to predict cognitive and functional decline in impaired participants was comparable to PET and CSF biomarkers of amyloid and tau. Lastly, with respect to tracking, only longitudinal changes in C-NODDI ADI and amyloid PET correlated with longitudinal changes in cognition and function.

Taken together, the above findings suggest that axonal loss is a key aspect of neurodegeneration in AD, as well as a major contributor to cognitive and functional outcomes. Notably, changes in ADI were evident in the prodromal mild cognitive impairment stage. Strengths of the study include its reliance on the robust ADNI dataset, longitudinal analyses, which fill a critical gap in current microstructure MRI literature, and comparison with established AD biomarkers. Some limitations of the study include its short follow-up period, which did not permit evaluation of preclinical outcomes, and that analyses were restricted to whole white matter, with the spatial pattern of ADI values not extensively evaluated.

## Clinical implications and future directions

This work has important implications for expanding the repertoire of clinically relevant markers of neurodegeneration that have prognostic value and for understanding the pathways to neurodegeneration in AD and related disorders. From a methodological perspective, it provides an easily implemented and clinically scalable solution for microstructure model instability in the presence of high free water infiltration, as regularly seen in AD. Future studies should explore other free water correction strategies that may further improve the sensitivity of ADI. The expense, equipment, and expertise required to collect diffusion MRI are a limitation to accessibility, particularly in resource-scarce environments. However, current developments in low-field MRI may alleviate these limitations in the future. From a pathophysiologic perspective, this work highlights that neurodegeneration is not a monolithic process in AD and that axonal injury is an important determinant and predictor of impairment that may serve as a critical metric for evaluating therapeutic interventions. As such, future work should focus on defining the value of measuring axonal injury during the preclinical stage, spatial variation in axonal injury across the brain, and the relationship of this variation to the heterogeneity of pathology that defines AD and related disorders. Overall, this work shines an important and innovative spotlight on neurodegeneration in AD of relevance to improved detection, monitoring, and therapeutic development.

## Funding support

This work is the result of NIH funding, in whole or in part, and is subject to the NIH Public Access Policy. Through acceptance of this federal funding, the NIH has been given a right to make the work publicly available in PubMed Central.

- NIH grant P30AG066512.
- Agence National de la Recherche grant ANR-22-CE17-0060.

## Version 1 02/02/2026

Electronic publication
